# Impact of a smartphone app on prescriber adherence to antibiotic guidelines in adult patients with community acquired pneumonia or urinary tract infections

**DOI:** 10.1371/journal.pone.0211157

**Published:** 2019-01-29

**Authors:** Chang Ho Yoon, Stephen R. Ritchie, Eamon J. Duffy, Mark G. Thomas, Stephen McBride, Kerry Read, Rachel Chen, Gayl Humphrey

**Affiliations:** 1 Auckland District Health Board, Grafton, Auckland, New Zealand; 2 School of Medical Sciences, University of Auckland, Grafton, Auckland, New Zealand; 3 Counties Manukau District Health Board, Otahuhu, Auckland, New Zealand; 4 Waitemata District Health Board, Takapuna, Auckland, New Zealand; 5 National Institute for Health Innovation, University of Auckland, Glen Innes, Auckland, New Zealand; Northwestern University Feinberg School of Medicine, UNITED STATES

## Abstract

**Background:**

Mobile phone apps have been shown to enhance guideline adherence by prescribers, but have not been widely evaluated for their impact on guideline adherence by prescribers caring for inpatients with infections.

**Objectives:**

To determine whether providing the Auckland City Hospital (ACH) antibiotic guidelines in a mobile phone app increased guideline adherence by prescribers caring for inpatients with community acquired pneumonia (CAP) or urinary tract infections (UTIs).

**Methods:**

We audited antibiotic prescribing during the first 24 hours after hospital admission in adults admitted during a baseline and an intervention period to determine whether provision of the app increased the level of guideline adherence. To control for changes in prescriber adherence arising from other factors, we performed similar audits of adherence to antibiotic guidelines in two adjacent hospitals.

**Results:**

The app was downloaded by 145 healthcare workers and accessed a total of 3985 times during the three month intervention period. There was an increase in adherence to the ACH antibiotic guidelines by prescribers caring for patients with CAP from 19% (37/199) to 27% (64/237) in the intervention period (p = 0.04); but no change in guideline adherence at an adjacent hospital. There was no change in adherence to the antibiotic guidelines by prescribers caring for patients with UTI at ACH or at the two adjacent hospitals.

**Conclusions:**

Provision of antibiotic guidelines in a mobile phone app can significantly increase guideline adherence by prescribers. However, providing an app which allows easy access to antibiotic guidelines is not sufficient to achieve high levels of prescriber adherence.

## Introduction

In response to the growing threat of antibiotic resistance, antibiotic stewardship programmes in primary and secondary care have introduced myriad prescribing and decision support tools in order to improve rates of appropriate antibiotic prescribing [1]. Whilst high levels of adherence to antibiotic guidelines result in improved patient safety, improved treatment outcomes, and reduced antibiotic resistance [1, 2], adherence to these guidelines often remains low [3–5]. Multiple factors are thought to contribute to this problem: difficulties with accessing the guidelines; prescribers’ lack of confidence in the processes used for guideline development; prescribers’ perceptions that their own expertise results in better treatment decisions than those suggested by the guidelines; and institutional healthcare cultures which support idiosyncratic prescribing behaviour [4, 6].

The deployment of computerized decision support (CDS) on non-mobile platforms has repeatedly been shown to improve adherence to antibiotic guidelines, reduce mortality rates, and decrease the prevalence of antibiotic resistance [2]. Despite this evidence, the provision and uptake of CDS at a health system level remains low [7]. Conversely, mobile health (mHealth) platforms are now ubiquitous within society. The growth in their provision and use has far exceeded the pace of their regulation, monitoring and research [8].

The widespread use of smartphones by healthcare workers has led to a proliferation of smartphone applications (apps), including many that have made antimicrobial guidelines readily available throughout the healthcare workplace [9]. Despite a dearth of evidence that access to these apps impacts on prescribing behaviour or patient outcomes, several studies have shown their widespread use in clinical practice, presumably because of the ease with which they facilitate point-of-care access to the guidelines at the time decisions are being made [10–12].

In light of the success of apps in improving adherence to guidelines for the treatment of depression and cardiac arrest [13, 14], it is logical to expect that apps can also improve prescriber adherence to antibiotic guidelines. To date, only one study has assessed the impact of an antibiotic guideline app on prescriber adherence. This study found that provision of an antimicrobial guideline app, which was co-designed, highly regarded, and widely downloaded by clinicians [12], was associated with a statistically significant increase in prescriber adherence to the hospital antimicrobial guidelines [15].

Recent studies that documented low rates of adherence to the Auckland City Hospital (ACH) antibiotic guidelines for the treatment of community acquired pneumonia (CAP)[16], and urinary tract infection (UTI; personal communication E. Duffy) prompted us to co-design, develop and implement an app (named SCRIPT) containing the ACH antibiotic guidelines. This study evaluated the impact of SCRIPT on prescriber adherence to antibiotic guidelines for CAP and UTI.

## Methods

We designed our study to test the hypothesis that the introduction of the SCRIPT app, which provides the ACH antibiotic guidelines in a user-friendly, decision-making process format, would increase prescriber adherence to these guidelines. We investigated the early impact of SCRIPT on the initial empiric treatment of adult patients admitted to ACH with either community-acquired pneumonia (CAP) or urinary tract infection (UTI). The study was approved by the New Zealand Health and Disabilities Ethics Committee (reference number: 16/STH/6).

### The intervention: SCRIPT

The existing ACH antibiotic guidelines were directly mapped into decision trees (e.g. “Does the patient have a history of allergy to penicillin?” → “Yes” or “No”), that branched out to the eventual antibiotic treatment recommendations.

**Fig 1:**
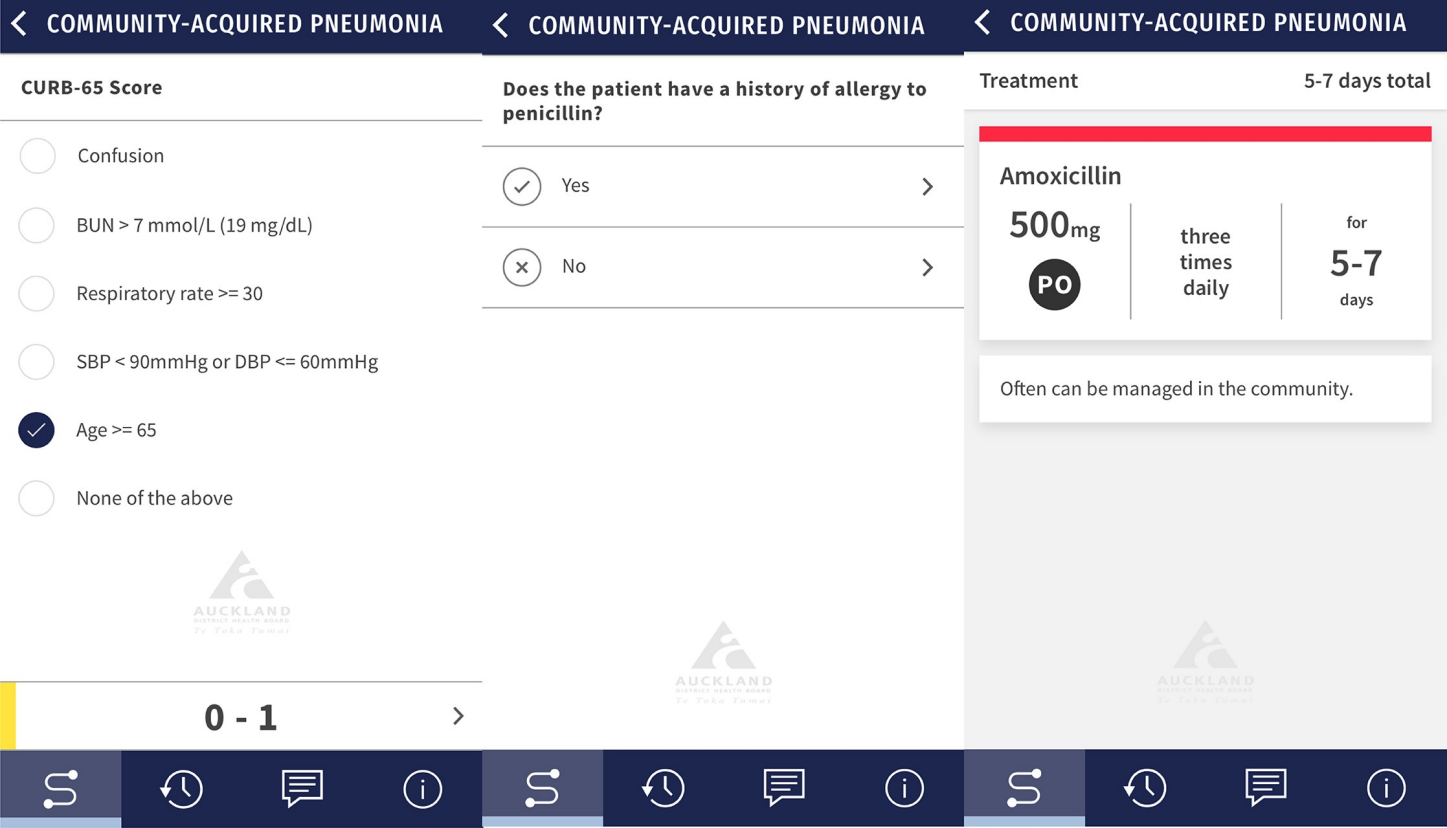
SCRIPT appearance and layout for a patient with mild CAP.

The user interface and functionality of the app was designed in collaboration with the Design for Health and Wellbeing Lab (ACH). Following a rigorous quality assurance process involving test users and clinicians from our research team, the ACH Antibiotic Stewardship Committee approved the app’s publication on the iOS and Android app stores. Fig 1 shows an example of SCRIPT’s layout and decision trees for mild CAP.

When accessing the app, users of SCRIPT consented to collection of anonymous data regarding their app usage via an in-built feature of the app.

### Study description

ACH, Middlemore Hospital (MMH) and North Shore & Waitakere Hospitals (NSH/WTH) serve a combined population of approximately 1.6 million people resident within the Auckland metropolis.

The study retrospectively measured prescriber adherence to the ACH antibiotic guidelines in adult patients (>18 years) with CAP (ICD-10 codes J10-18, 22) or UTI (ICD-10 codes N10-12, 30, 39), who had been admitted to ACH for ≥4 hours during baseline and intervention periods in 2016. The initial antibiotic prescription(s) in the first 24 hours of admission for patients admitted during the baseline period (1 January 2016 to 31 May 2016), before SCRIPT was released, was compared with that for patients admitted during the intervention period (1 June 2016 to 31 August 2016) after SCRIPT had been made available for all ACH prescribers. Hospital data from the previous three years indicated that a three month period would include an appropriate number of patients (200) to provide sufficient power to detect our estimate of SCRIPT’s impact.

To allow for potential confounding by other concurrent factors, we also measured prescriber adherence to the relevant antibiotic guidelines in adult patients admitted for ≥4 hours to MMH and NSH/WTH during the same baseline and intervention periods. SCRIPT was introduced from 1 June 2016, coinciding with both the beginning of a junior doctor rotation, and the beginning of the intervention period. Since junior doctors may move between these regional hospitals on 3-monthly rotations, the study periods intentionally coincided with the beginning of a junior doctor rotation. In the first two weeks of the intervention period, educational sessions, posters and intranet advertisements were employed to socialize the app and facilitate its uptake by clinicians at ACH.

Antibiotic stewardship activities at MMH and NSH/WTH, which uphold the same principles as those at ACH, continued during the study period, but no major interventions to improve the management of CAP or UTI were undertaken. To prevent app usage by clinicians in MMH and NSH/WTH, SCRIPT was restricted to ACH clinicians through unique activation passcodes linked to active ACH e-mail accounts. In all of the hospitals, antibiotic guidelines remained accessible through their respective pocket guides, posters, and intranet sites. Electronic prescribing and other decision support tools were not introduced during the study period.

### Study participants

We calculated that samples of 200 cases of CAP and 200 cases of UTI admitted to ACH during the baseline and intervention periods would be required to achieve 90% power to detect an absolute 15% improvement in prescriber adherence to antibiotic guidelines at ACH (α = 0.05). We therefore measured guideline adherence in over 200 randomly selected cases of CAP and UTI, during the baseline and intervention periods, at ACH, MMH and NSH/WTH. We then compared changes in levels of guideline adherence at ACH with those at MMH and NSH/WTH to determine whether any changes in prescriber adherence at ACH could confidently be ascribed to the introduction of the SCRIPT app.

Patients were excluded if, on review of their records, they were not diagnosed with CAP or UTI during the first 24 hours of their admission; or if their diagnosis was miscoded (e.g. “empyema” coded as CAP, “renal colic” coded as UTI); or if they were transferred to the hospital from another secondary care facility where antibiotic treatment had already been initiated.

### Data collection

Manual data collection and system level data extraction were used to collect study data. A case report form (CRF) was developed using REDCap (Nashville, Tennessee) data capture tools hosted at the University of Auckland; two research pharmacists completed a CRF for each randomly selected episode. For patients admitted to ACH, antibiotic details (name, dose, route, frequency during the first 24 hours of admission) were collected using the CRF. For patients admitted to the other hospitals, antibiotic data was collected by system level data extraction from the relevant eMedicines systems (ePrescribing and/or Pyxis medicine cabinets). The same system level data extraction methods were used for demographic and admission data (e.g. age, ethnicity, gender, length of stay, 28-day all-cause mortality). Manual data collection was necessary for clinical data (e.g. the diagnostic impression, chest X-ray reports, CURB-65 variables, urinalysis results, presence of fever and flank pain).

Data quality was ensured by independent re-collection and review of 10% of the data. Any interpretative uncertainties of clinical data were resolved by review and discussion by at least two study clinicians. For example, a chest X-ray report stating “there is increased air space opacity in the right lower lobe” was interpreted as “consolidation” (pneumonia).

### Analysis and definitions of adherence and appropriate treatment

The clinician’s diagnosis and other relevant information documented in each case’s notes, during the first 24 hours of their admission, were used to determine whether treatment given during this period was guideline adherent. For cases with an otherwise unspecified diagnosis of “urinary tract infection”, we retrospectively considered all patients with flank pain or hypotension to have had pyelonephritis, and those with neither of these features to have had cystitis [17].

We defined management as adherent if the antibiotic(s) prescribed, and their dose(s) and route(s) of administration, were consistent with the relevant hospital guidelines. Cases that had been prescribed a guideline-adherent antibiotic regimen during the first 24 hours of their admission were considered to be adherent regardless of whether, or not, they had also received other guideline non-adherent regimen(s) (e.g. prescribed by a different clinician) during the first 24 hours.

We defined appropriate treatment as any treatment that was neither over-treatment nor under-treatment. We defined over-treatment as prescription of inappropriately broad-spectrum agents. For example, prescription of cefuroxime plus erythromycin for a patient with an admission diagnosis of CAP and a CURB-65 score of 1 was considered overtreatment. We defined under-treatment as prescription of inappropriately narrow-spectrum or ineffective agents. For example, prescription of trimethoprim or nitrofurantoin for a patient with an admission diagnosis of pyelonephritis was considered under-treatment.

For cases with a diagnosis of CAP documented in their notes during the first 24 hours of admission, but without a documented CURB-65 score, we calculated the score using the relevant clinical data. For cases without a serum urea result, we calculated the CRB-65 score [18]. Management of cases with a CRB-65 score of 0 or 3–4 was considered adherent if the antibiotics prescribed were those recommended for patients with a CURB-65 score of 0–1 or 3–5, respectively. Management of cases with a CRB-65 score of 1 was considered adherent if the antibiotics prescribed were those recommended for patients with CURB-65 scores of 0–1 or 2. Similarly, the management of cases with CRB-65 score of 2 was considered adherent if the antibiotics prescribed were those recommended for patients with CURB-65 scores of 2 or 3–5.

We used IBM SPSS (Armonk, New York) version 25 to analyse all data. Continuous variables were reported as medians with interquartile ranges (IQR). Categorical variables were expressed as numbers or percentages. The baseline and intervention period rates of adherence and appropriate treatment were compared with Pearson’s chi-squared test.

The guidelines for management of CAP in NSH/WTH depended upon the SMART-COP pneumonia severity score [19], while the ACH and MMH guidelines relied upon the CURB-65 pneumonia severity score [18]. Therefore, antibiotic guideline adherence for patients with CAP admitted to NSH/WTH was not compared with ACH.

## Results

Only 53 prescribers downloaded SCRIPT during the first month of the intervention period. This number rose gradually to 145 healthcare workers by the end of the study and the respiratory guidelines were the most frequently accessed during the study(Table 1).

**Table 1:** Numbers of healthcare workers viewing the SCRIPT app for each body system during the intervention period in 2016.

Body System	June	July	August	Total
Respiratory	218	379	250	847
Genitourinary	86	157	107	350
Sepsis	128	121	218	467
CNS	64	90	124	278
Ophthalmology	71	54	89	214
ENT	120	87	128	335
Cardiothoracic	48	29	37	114
Gastrointestinal tract	112	136	252	500
Bone and joint	66	64	87	217
Skin and soft tissue	184	249	230	663
Total	1097	1366	1522	3985

The approximate average number of times that SCRIPT guidelines were accessed by each user was 21 in June (1097/53), 12 in July (1366/112) and 11 in August (1522/145). The median time spent using the CAP guideline on SCRIPT was 11 seconds (IQR 7–17) and the median time spent using the UTI guideline was 18 seconds (IQR 12–29).

### Antibiotic guideline adherence for patients with community acquired pneumonia

Most of the demographic and clinical features of the CAP cases did not differ significantly between the baseline and intervention cohorts at ACH (Table 2). However, the clinical diagnosis of “viral respiratory tract infection” was significantly more common in the ACH intervention cohort than in the ACH baseline cohort (chi-squared, p<0.001).

**Table 2:** Demographic and clinical features (n (%)) of patients with CAP admitted to ACH or MMH during the baseline and intervention periods.

	ACH		MMH	
Features	Baseline n = 199	Intervention n = 237	Baseline n = 211	Intervention n = 209
Median age (IQR), years	62 (46–77)	64 (44–79)	71 (60–79)	66 (50–76)
Female gender	96 (48)	139 (59)	93 (44)	118 (57)
Ethnicity*				
European	90 (45)	121 (51)	79 (37)	75 (36)
Māori	22 (11)	36 (15)	58 (28)	48 (23)
Pacific Islander	40 (20)	48 (20)	62 (29)	59 (28)
Asian / Other	47 (24)	32 (14)	12 (6)	27 (13)
CXR performed	196 (99)	235 (99)	209 (99)	209 (100)
CXR consolidation*	62 (31)	77 (33)	172 (82)	169 (81)
Diagnostic impression				
Pneumonia*	70 (35)	70 (30)	186 (88)	166 (79)
LRTI unspecified	107 (54)	98 (41)	18 (9)	29 (14)
Viral	18 (9)	61 (26)**	2 (1)	4 (2)
Bronchitis	2 (1)	2 (1)	0 (0)	2 (1)
Other	2 (1)	6 (3)	5 (2)	8 (4)
CURB-65 documented	31 (16)	50 (21)	42 (20)	37 (18)
C(U)RB-65***				
Score 0–1*	129 (65)	151 (64)	79 (37)	122 (58)
Score 2*	42 (21)	55 (23)	60 (28)	46 (22)
Score 3–5*	28 (14)	31 (13)	72 (34)	41 (20)
Median LOS days*	2	2	4	3
ICU admission	2 (1)	3 (1)	0 (0)	7 (3)
28-day mortality*	3 (2)	1 (1)	46 (22)	25 (12)

* p<0.05

** p<0.001

***The C(U)RB-65 scores were either recorded in the doctors’ notes or calculated from the clinical data.

ACH, Auckland City Hospital; MMH, Middlemore Hospital; CAP, community-acquired pneumonia; CXR, chest X-ray; LRTI, lower respiratory tract infection; LOS, length of stay; ICU, intensive care unit.

Differences were apparent between the ACH and MMH CAP cohorts with regard to ethnicity, the presence of consolidation on CXR, the proportion of patients with a clinical diagnosis of pneumonia, the CURB-65 scores, the median length of stay, and the mortality rate (chi-squared, all p<0.05). The populations served by these two hospitals differ considerably in terms of socio-economic status, with a higher prevalence of deprivation observed in the MMH population [20]. 160/856 (19%) of the total ACH and the MMH baseline and intervention cohorts had a CURB-65 score documented in their clinical records. A CURB-65 score could be determined for a further 576 cases and a CRB-65 score for 120 cases.

37/199 (19%) patients with CAP at ACH were prescribed guideline adherent treatment during the baseline period, and this improved to 64/237 (27%) during the intervention period (*p* = 0.04). There was a marked increase in adherence in the 59 patients with severe pneumonia (CURB-65 3–5), from 7% in the baseline period to 29% in the intervention period (p = 0.03). There was also a significant increase in the prescription of appropriate treatment at ACH, from 44/199 (22%) during the baseline period to 77/237(32%) during the intervention period (p = 0.02). In contrast, at MMH, there was no change in the rates of guideline adherence (19/211, 9% during the baseline period and 19/209, 9% during the intervention period, p = 0.98) or in the prescription of appropriate treatment (20/211, 9% during the baseline period and 21/209 (10%) during the intervention period, p = 0.84).

### Urinary tract infection

The demographic and clinical features of the UTI cases did not differ significantly between the baseline and intervention cohorts for ACH, MMH or NSH/WTH. There were significant differences between the ACH, MMH and NSH/WTH UTI cohorts with regard to age, gender, ethnicity, the presence of an indwelling urinary catheter, the presence of impaired renal function, and the 28-day mortality rate (chi-squared, p<0.05), reflecting the differences in the populations served by these hospitals (Table 3). In a total of 803 (64%) cases admitted to ACH, MMH or NSH/WTH during the baseline or intervention periods, the clinical records for the first 24 hours did not specify whether the clinical impression was specifically “cystitis” or “pyelonephritis”. We used the clinical data to retrospectively ascribe an admission diagnoses of either cystitis (n = 673) or pyelonephritis (n = 130).

**Table 3:** Demographic and clinical features (n(%)) of patients with UTI admitted to ACH, MMH and NSWH during the baseline and intervention periods.

	ACH		MMH		NSWH	
Features	Baseline n = 209	Intervention n = 211	Baseline n = 211	Intervention n = 201	Baseline n = 211	Intervention n = 206
Median age (IQR), years*	58 (30–79)	59 (34–78)	67 (42–78)	68 (46–82)	72 (48–84)	72 (43–82)
Female gender*	154 (74)	155 (74)	141 (67)	133 (66)	143 (68)	132 (64)
Ethnicity*						
European	113 (54)	122 (58)	89 (42)	93 (46)	166 (79)	161 (78)
Māori	17 (8)	32 (15)	41 (19)	42 (21)	13 (6)	18 (9)
Pacific Islander	21 (10)	18 (9)	46 (22)	41 (20)	17(8)	12 (6)
Asian / Other	58 (28)	39 (19)	35 (17)	25 (12)	15 (7)	15 (7)
Diagnosis documented						
Pyelonephritis	71 (34)	78 (37)	69 (33)	56 (28)	31 (15)	84 (41)
Cystitis	15 (7)	4 (2)	8 (4)	16 (8)	6 (3)	8 (4)
UTI not specified	123 (59)	129 (61)	134 (63)	129 (64)	174 (82)	114 (55)
Diagnosis						
Pyelonephritis	93 (44)	95 (45)	92 (44)	70 (35)	65 (31)	104 (51)
Cystitis	116 (56)	116 (55)	119 (56)	131 (65)	146 (69)	102 (49)
In-dwelling catheter*	9 (4)	13 (6)	25 (12)	22 (11)	25 (12)	36 (18)
eGFR < 30 ml/min/1.73m^2^ *	16 (8)	10 (5)	27 (13)	22 (11)	16 (8)	17 (8)
Pyuria	192 (92)	194 (92)	206 (98)	192 (96)	203 (96)	202 (98)
Median LOS, days	2	1	2	2	3	2
ICU admission	2 (1)	0 (0)	1 (0.5)	2 (1)	1 (0.5)	2 (1)
28-day mortality*	1 (0.5)	2 (1)	18 (9)	16 (8)	16 (8)	6 (3)

* p<0.05.

ACH = Auckland City Hospital; MMH = Middlemore Hospital; NSWH = North Shore & Waitakere Hospitals; UTI = urinary tract infection; eGFR = estimated glomerular filtration rate; LOS = length of stay; ICU = intensive care unit.

Guideline adherence for patients with UTI at ACH did not change significantly between the baseline period and the intervention period. During the baseline period 98/209 (47%) patients were prescribed guideline adherent treatment and during the intervention period 106/211 (50%) patients were prescribed guideline treatment (p = 0.49). At MMH (95/211 (45%) during the baseline period and 80/201 (40%) during intervention period, p = 0.28) and NSH/WTH (50/211 (24%) during the baseline period and 59/206 (29%) during the intervention period, p = 0.25), there were no significant changes in the rate of guideline adherence between the baseline and intervention periods. Furthermore, there were no significant changes in the prescription of appropriate treatment between the baseline and intervention periods at any of the hospitals.

## Discussion

The introduction of the SCRIPT antibiotic guideline app at ACH had a statistically significant, positive impact on prescriber adherence to the ACH antibiotic guidelines in patients with CAP. The rate of antibiotic guideline adherence improved by an absolute value of 8%, a relative increase of 42%, despite relatively slow uptake of the app. Importantly, in CAP, the introduction of SCRIPT was associated with a statistically significant rise in appropriate treatment (reductions in both over-treatment and in under-treatment). Whilst over-treatment with unnecessarily broad-spectrum antibiotics may cause harm by accelerating the development of antibiotic resistance, the potential consequences of under-treatment can be more immediately detrimental to patients. Although the 30-day mortality in the patients with CAP was not significantly reduced during the intervention period, this study was not designed to detect a significant change in patient outcomes, such as 30-day mortality or the rate of hospital readmission; larger studies would be required to answer such questions.

Our study is consistent with a recent meta-analysis of antibiotic stewardship measures, which concluded that point-of-care information is beneficial for stewardship [1]. Moreover, our findings are similar to the only comparable study of the impact of providing antibiotic guidelines in a mobile phone app, which found a 6.5% increase in guideline adherence, using an interrupted time series study [15]. It is unusual for interventions that are delivered via a single modality to improve antibiotic adherence. Clinical decision support interventions, typically delivered via desktop computers, have resulted in 1.5–61% increases in adherence in multiple studies, but have often required longer intervention periods of 6–12 months to achieve such effects [2].

Our study is consistent with many others that have shown how difficult it is to change prescriber behaviour [21–23]. The results of our study strongly suggest that, in general, the failure of clinicians to follow the ACH treatment guidelines was not the consequence of clinicians accessing the guidelines and then actively deciding to choose another treatment regimen, but instead the failure of clinicians to access and consider the guidelines at all. If clinicians caring for patients with CAP at ACH had accessed and considered the local guidelines, it seems likely that the rate of documentation of the CURB-65 score, on which the guideline is dependent, would have been higher than 16% in the baseline period and 21% in the intervention period. A similar conclusion can be drawn from the consistently high proportion of cases with UTI at ACH (59% in the baseline period and 61% in the intervention period), in whom the treating clinicians did not indicate whether treatment was for cystitis or pyelonephritis, despite the guideline’s dependence on discriminating between these two conditions before generating a treatment recommendation.

The reasons for the marked difference between CAP and UTI in the rates of guideline adherence are not immediately clear. Low rates of CURB-65 score documentation were paralleled by low rates of documentation of specific UTI diagnoses. The UTI decision tree in SCRIPT has a minimum of six branch points, compared with only three for CAP; this results in more questions and decision points, before the clinician using the app obtains the treatment recommendation. Whilst this was due to the complexity of both establishing the UTI diagnosis and the consideration of other factors (such as the presence of renal failure and colonization by extended spectrum beta-lactamase producing bacteria), it is difficult to ascertain if this was a deterrent to SCRIPT use as there was little difference in the average time taken to access the SCRIPT recommendation (CAP 20 seconds; UTI 23 seconds).

The frequency of accessing SCRIPT was high in comparison with a study conducted in London, reported by Charani, et al [15]. In that study, the proportion of doctors at two London hospitals who downloaded their antibiotic guideline app was 61% at one hospital, and 100% at the other; however, the average rate of use of the app was less than twice per month at both hospitals [10, 24]. In contrast, the average rate of SCRIPT use was five times higher. Taken together these combined results indicate that improving prescriber adherence to treatment guidelines is considerably more complex than simply facilitating access to the guidelines [10, 12, 24]. A systematic review of antibiotic prescribing for respiratory tract infections has shown that prescribing practices are complex and include factors such as prior knowledge and experience, as well as perceptions about prevention of adverse outcomes [25].

In summary we found that the introduction of a smartphone app, that made the ACH antibiotic guidelines readily available to all ACH clinicians, was associated with a statistically significant increase in prescriber adherence to the guidelines in patients with CAP. While it is likely that the full impact of SCRIPT would only be observed after the app has become embedded into clinical practice over a longer time period, our study shows that release of an antibiotic guideline app will lead to almost immediate improvements. However, we also found that, despite high usage of SCRIPT, clinicians very commonly failed to perform those assessments necessary to determine the optimum treatment, most importantly through measuring the severity of illness in patients with CAP (by use of the CURB-65 score) or deciding on the site of infection in patients with a UTI (pyelonephritis or cystitis). Improvements in the care of patients with common infections requires more than making guidelines readily available.

SCRIPT has continued to be promoted since the completion of this study, and up to June 2018 there have been over 3000 downloads within ACH. We intend to perform further studies of the temporal changes in the level of prescriber adherence; and the reasons given by clinicians for failure to use the guideline and adhere to its evidence based recommendation.

## References

[1] Davey P, Marwick CA, Scott CL, Charani E, McNeil K, Brown E, et al Interventions to improve antibiotic prescribing practices for hospital inpatients. Cochrane Database of Syst Rev. 2017(2). 10.1002/14651858.CD003543.pub4 PMC6464541 28178770

[2] Curtis CE, Al Bahar F, Marriott JF. The effectiveness of computerised decision support on antibiotic use in hospitals: A systematic review. PLoS One. 2017;12(8):e0183062 10.1371/journal.pone.0183062 28837665 PMC5570266

[3] Schuts EC, Hulscher MEJL, Mouton JW, Verduin CM, Stuart JWTC, Overdiek HWPM, et al Current evidence on hospital antimicrobial stewardship objectives: a systematic review and meta-analysis. Lancet Infect Dis. 2016;16(7):847–56. 10.1016/S1473-3099(16)00065-7 26947617

[4] Mol PGM, Rutten WJMJ, Gans ROB, Degener JE, Haaijer-Ruskamp FM. Adherence Barriers to Antimicrobial Treatment Guidelines in Teaching Hospital, the Netherlands. Emerg Infect Dis. 2004;10(3):522–5. 10.3201/eid1003.030292 15109428 PMC3322770

[5] Dellit TH, Owens RC, McGowan JE, Gerding DN, Weinstein RA, Burke JP, et al Infectious Diseases Society of America and the Society for Healthcare Epidemiology of America Guidelines for Developing an Institutional Program to Enhance Antimicrobial Stewardship. Clin Infect Dis. 2007;44(2):159–77. 10.1086/510393 17173212

[6] Charani E, Castro-Sanchez E, Sevdalis N, Kyratsis Y, Drumright L, Shah N, et al Understanding the Determinants of Antimicrobial Prescribing Within Hospitals: The Role of “Prescribing Etiquette”. Clin Infect Dis. 2013;57(2):188–96. 10.1093/cid/cit212 23572483 PMC3689346

[7] Ahmed Z, McLeod MC, Barber N, Jacklin A, Franklin BD. The Use and Functionality of Electronic Prescribing Systems in English Acute NHS Trusts: A Cross-Sectional Survey. PLoS One. 2013;8(11):e80378 10.1371/journal.pone.0080378 24278279 PMC3835329

[8] Charani E, Castro-Sánchez E, Moore LS, Holmes A. Do smartphone applications in healthcare require a governance and legal framework? It depends on the application! BMC Med. 2014;12(1):29. 24524344 10.1186/1741-7015-12-29 PMC3929845

[9] Batista MA, Gaglani SM. The future use of smartphones in healthcare. Virtual Mentor. 2013;15(11):947–50. 10.1001/virtualmentor.2013.15.11.stas1-1311 24257085

[10] Panesar P, Jones A, Aldous A, Kranzer K, Halpin E, Fifer H, et al Attitudes and Behaviours to Antimicrobial Prescribing following Introduction of a Smartphone App. PLoS One. 2016;11(4):e0154202 10.1371/journal.pone.0154202 27111775 PMC4844117

[11] Markley JD, Pakyz A, Bernard S, Lee K, Appelbaum N, Bearman G, et al A survey to optimize the design of an antimicrobial stewardship smartphone app at an academic medical center. Am J Infect Control. 2017;45(3):317–20. 10.1016/j.ajic.2016.09.026 27838166

[12] Charani E, Kyratsis Y, Lawson W, Wickens H, Brannigan ET, Moore LSP, et al An analysis of the development and implementation of a smartphone application for the delivery of antimicrobial prescribing policy: lessons learnt. J Antimicrob Chemother. 2013;68(4):960–7. 10.1093/jac/dks492 23258314 PMC3594497

[13] Trivedi MH, Kern JK, Grannemann BD, Altshuler KZ, Sunderajan P. A Computerized Clinical Decision Support System as a Means of Implementing Depression Guidelines. Psychiatr Serv. 2004;55(8):879–85. 10.1176/appi.ps.55.8.879 15292537

[14] Field LC, McEvoy MD, Smalley JC, Clark CA, McEvoy MB, Rieke H, et al Use of an Electronic Decision Support Tool Improves Management of Simulated In-Hospital Cardiac Arrest. Resuscitation. 2014;85(1):138–42. 10.1016/j.resuscitation.2013.09.013 24056391 PMC4116642

[15] Charani E, Gharbi M, Moore LSP, Castro-Sanchéz E, Lawson W, Gilchrist M, et al Effect of adding a mobile health intervention to a multimodal antimicrobial stewardship programme across three teaching hospitals: an interrupted time series study. J Antimicrob Chemother. 2017;72(6):1825–31. 10.1093/jac/dkx040 28333297 PMC5437525

[16] Aikman KL, Hobbs MR, Ticehurst R, Karmakar GC, Wilsher ML, Thomas MG. Adherence to Guidelines for Treating Community-Acquired Pneumonia at a New Zealand Hospital. Journal of Pharmacy Practice and Research. 2013;43(4):272–5.

[17] Gupta K, Hooton TM, Naber KG, Wullt B, Colgan R, Miller LG, et al International Clinical Practice Guidelines for the Treatment of Acute Uncomplicated Cystitis and Pyelonephritis in Women: A 2010 Update by the Infectious Diseases Society of America and the European Society for Microbiology and Infectious Diseases. Clin Infect Dis. 2011;52(5):e103–e20. 10.1093/cid/ciq257 21292654

[18] Lim WS, van der Eerden MM, Laing R, Boersma WG, Karalus N, Town GI, et al Defining community acquired pneumonia severity on presentation to hospital: an international derivation and validation study. Thorax. 2003;58(5):377 10.1136/thorax.58.5.377 12728155 PMC1746657

[19] Charles PGP, Wolfe R, Whitby M, Fine MJ, Fuller AJ, Stirling R, et al SMART-COP: A Tool for Predicting the Need for Intensive Respiratory or Vasopressor Support in Community-Acquired Pneumonia. Clin Infect Dis. 2008;47(3):375–84. 10.1086/589754 18558884

[20] Walls G, Vandal AC, du Plessis T, Playle V, Holland DJ. Socioeconomic factors correlating with community antimicrobial prescribing. NZ Med J. 2015;128(1417):16–23. 26149899

[21] Sbarbaro JA. Can We Influence Prescribing Patterns? Clin Infect Dis. 2001;33(Supplement_3):S240–S4. 11524726 10.1086/321856

[22] O'Brien MA, Rogers S, Jamtvedt G, Oxman AD, Odgaard-Jensen J, Kristoffersen DT, et al Educational outreach visits: effects on professional practice and health care outcomes. Cochrane Database of Syst Rev. 2007(4). 10.1002/14651858.CD000409.pub2 PMC7032679 17943742

[23] Brennan N, Mattick K. A systematic review of educational interventions to change behaviour of prescribers in hospital settings, with a particular emphasis on new prescribers. Br J Clin Pharmacol. 2012;75(2):359–72. 10.1111/j.1365-2125.2012.04397.x PMC3579251 22831632

[24] Cabana MD, Rand CS, Powe NR, et al Why don't physicians follow clinical practice guidelines? A framework for improvement. JAMA. 1999;282(15):1458–65. 10535437 10.1001/jama.282.15.1458

[25] McKay R, Mah A, Law MR, McGrail K, Patrick DM. Systematic Review of Factors Associated with Antibiotic Prescribing for Respiratory Tract Infections. Antimicrob Agents Chemother. 2016;60(7):4106–18. 10.1128/AAC.00209-16 27139474 PMC4914667

